# Droplet digital PCR is a powerful technique to demonstrate frequent *FGFR1* duplication in dysembryoplastic neuroepithelial tumors

**DOI:** 10.18632/oncotarget.12881

**Published:** 2016-10-25

**Authors:** Frédéric Fina, Doriane Barets, Carole Colin, Corinne Bouvier, Laëtitia Padovani, Isabelle Nanni-Metellus, L’Houcine Ouafik, Didier Scavarda, Andrey Korshunov, David T.W. Jones, Dominique Figarella-Branger

**Affiliations:** ^1^ Assistance Publique Hôpitaux de Marseille (AP-HM), Hôpital Nord, Service de Transfert d'Oncologie Biologique, Laboratoire de Biologie Médicale Marseille, France; ^2^ APHM, Hôpital de la Timone, Service d’Anatomie Pathologique et de Neuropathologie, Marseille, France; ^3^ Aix-Marseille Université, Inserm, CRO2 UMR_S 911, Marseille, France; ^4^ APHM, Hôpital de la Timone, Service de Radiothérapie, Marseille, France; ^5^ APHM, Hôpital de la Timone, Service de Neurochirurgie Pédiatrique, Marseille, France; ^6^ Division of Pediatric Neurooncology, German Cancer Research Center (DKFZ), Heidelberg, Germany

**Keywords:** dysembryoplastic neuroepithelial tumor (DNT), low grade neuroepithelial tumor (LGNT), FGFR1, droplet digital PCR (DDPCR™), MAP kinase pathway

## Abstract

Dysembryoplastic neuroepithelial tumors (DNT) share V600E mutation in the *BRAF* gene with other low grade neuroepithelial tumors (LGNTs). *FGFR1* internal tandem duplication of the tyrosine-kinase domain (*FGFR1*-ITD), another genetic alteration that also leads to MAP kinase pathway alteration, has been previously reported in LGNTs by whole-genome sequencing. In the present study we searched for *FGFR1*-ITD by droplet digital PCR (DDPCR™) and for *FGFR1* point mutations by HRM-sequencing in a series of formalin-fixed paraffin-embedded (FFPE) LGNTs including 12 DNT, 2 oligodendrogliomas lacking *IDH* mutation and 1p/19q co- deletion (pediatric-type oligodendrogliomas; PTOs), 3 pediatric diffuse astrocytomas (PDAs), 14 gangliogliomas (GGs) and 5 pilocytic astrocytomas (PAs). We showed by DDPCR™ that 5/12 DNT, but none of the other LGNTs, demonstrated *FGFR1*-ITD. In addition, these cases also accumulated phosphorylated-FGFR1 protein as shown by immunohistochemistry. FGFR1^G539R^ point mutation was only recorded in one DNT that also showed *FGFR1*-ITD. Interestingly, these *FGFR1* alterations were mutually exclusive from *BRAF^V600E^* mutation that was recorded in 13 LGNTs (3 DNTs, 1 PTO, 2 PDAs, 5 GGs and 2 PAs). Therefore, *FGFR1* alteration mainly represented by *FGFR1*-ITD is a frequent event in DNT. DDPCR™ is an easy and alternative method than whole-genome sequencing to detect *FGFR1*-ITD in FFPE brain tumors, in routine practice.

## INTRODUCTION

Dysembryoplastic neuroepithelial tumors (DNTs) are benign cortical tumors often occurring in the context of refractory epilepsy in children and young adults [1, 2] Two main histological forms of DNT have been described and are recognized by the 2016 WHO classification [3, 4]: 1/ the complex form and 2/ the simple form restricted to the glioneuronal element (GNE). The non-specific form that does not show the GNE but displays the same neuroimaging features as complex DNT is a highly controversial issue. These pediatric tumors have histological features of diffuse astrocytic and oligodendroglial tumors but never demonstrate *IDH* mutation nor 1p/19q co-deletion [5]. In the 2016 WHO classification, they are named pediatric diffuse astrocytoma (PDA) and oligodendroglioma lacking *IDH*-mutation and 1p/19q codeletion (pediatric-type oligodendroglioma; PTO) respectively [4]. It is worth noticing that in the previous version of the WHO classification of the central nervous system tumors [6], pediatric diffuse gliomas were grouped with their adult counterparts and classified in the same way.

We and others have recently reported V600E mutation of the *BRAF* gene in up to 20% of DNTs [7]. The MAP kinase pathway plays a major role in signal transduction and can be activated through a cascade of enzymatic reactions. Outside-in signaling can occur through the activation of membranous cell surface receptors displaying intracytoplasmic tyrosine kinase domains (TKD). The FGFR1 receptor belongs to this class of molecules [8]. Some recurrent aberrations affecting *FGFR1* have been reported in pediatric brain tumors including hotspot point mutations and a novel internal duplication of the kinase domain termed TKD-duplicated or FGFR1 internal tandem duplication (*FGFR1*-ITD). Another infrequent *FGFR1*aberration that has been reported is the *FGFR1-TACC1* fusion [9]. All these *FGFR1* molecular alterations are predicted to result in constitutive FGFR signaling. The *FGFR1*-ITD has first been reported in 1 case of PA by Jones and coworkers [10] and simultaneously in 13 LGNTs including 2 other PAs, 4 oligoastrocytomas, 3 diffuse astrocytomas, 3 oligodendrogliomas and 1 DNT by Zhang and coworkers [11]. This second group has recently confirmed these results in a large study [12]. However, in these three recent reports [10–12], LGNTs were analyzed by whole-genome sequencing using frozen specimen, which is not suitable for routine diagnostic practice.

In the present study, our aim was to set up an easy and highly sensitive test using the droplet digital PCR (DDPCR™) in order to detect *FGFR1*-ITD by using very small amount of formalin-fixed paraffin-embedded (FFPE) tissue in routine practice and to analyze the frequency of this alteration in pediatric LGNTs. To do so, we selected a series of 36 pediatric cases (12 DNTs, 2 PTOs, 3 PDAs, 14 GGs and 5 PAs) in which *BRAF*^V600E^ status was known [7]. All the DNTs, PTOs and PDAs lacked *IDH* mutation and 1p/19q co-deletion [5].

## RESULTS

Clinical and biological characteristics of the 36 patients, already reported in a previous paper for 35/36 [7], are summarized in Table 1.

**Table 1:** Clinical and biological characteristics of the 36 patients

Patient	Age at diagnosis (years)	Gender	Tumor location	Surgical resection	BRAF mutation status [4]	FGFR1 duplication (DDPCR™)	FGFR1 status by IHC	FGFR1 exon 12 mutation status	FGFR1 exon 14 mutation status	Progression	Overall survival (months)	Status at last follow-up
DNT18	7	m	temporal	NA	Non-mutated	No	+/-	NA	NA	Yes	164.4	NA
DNT17	5	f	temporal	Complete	V600E	No	Negative	Non-mutated	Non-mutated	No	51.7	FOD
DNT12	12	m	temporal	Complete	V600E	No	Negative	Non-mutated	NA	No	100	FOD
DNT07	9	f	temporal	Complete	Non-mutated	No	Negative	Non-mutated	Non-mutated	Yes	186.8	FOD
DNT04	13	m	temporal	Complete	Non-mutated	No	Negative	NA	NA	No	6.1	NA
DNT19	15	m	frontal	Complete	Non-mutated	No	Negative	NA	NA	No	83.8	FOD
DNT11	13	m	temporal	Complete	V600E	Inconclusive	Negative	Non-mutated	NA	No	67.5	FOD
DNT20	15	f	temporal	Partial	Non-mutated	Yes	Positive	G539R	Non-mutated	Yes	95.6	FOD
DNT14	10	f	occipital	Complete	Non-mutated	Yes	Positive	Non-mutated	NA	No	96	FOD
DNT03	15	m	frontal	Complete	Non-mutated	Yes	NA	Non-mutated	NA	No	36.4	NA
DNT21	3	m	frontal	NA	Non-mutated	Yes	Positive	Non-mutated	Non-mutated	No	54	FOD
DNT10*	11	m	temporal	Complete	Non-mutated	Yes	Positive	Non-mutated	Non-mutated	No	97.2	FOD
PTO01	16	m	parietal	Complete	Non-mutated	No	+/–	NA	NA	Yes	135.4	FOD
PTO02	17	f	temporal	Partial	V600E	No	+/–	NA	NA	No	69.1	FOD
PDA01	11	m	temporal	Complete	Non-mutated	No	Negative	NA	NA	Yes	119.9	FOD
PDA02	0	m	temporal	Partial	V600E	No	Negative	Non-mutated	Non-mutated	No	72.9	FOD
PDA03	12	f	temporal	NA	V600E	No	Negative	Non-mutated	Non-mutated	No	60.3	FOD
GG04*	11	m	temporal	Complete	Non-mutated	No	+/–	NA	NA	No	109	FOD
GG30	14	m	brainstem + spinal cord	Partial	V600E	No	Negative	NA	NA	Yes	170.9	AWSD
GG11*	14	m	frontal	Complete	V600E	No	Negative	Non-mutated	Non-mutated	No	55.1	FOD
GG21	12	f	parietal	Complete	Non-mutated	No	Negative	Non-mutated	Non-mutated	No	32.7	AWSD
GG09	1	f	brainstem	Partial	Non-mutated	No	Negative	Non-mutated	Non-mutated	No	122.2	FOD
GG22	1	m	parietal + occipital	Complete	Non-mutated	No	+/–	NA	NA	No	217.5	NA
GG06	6	m	spinal cord	Complete	V600E	No	Negative	Non-mutated	Non-mutated	No	116.3	FOD
GG14	12	m	temporal	Partial	Non-mutated	No	+/–	L548L	NA	No	57.9	FOD
GG12	13	m	temporal	Complete	Non-mutated	No	+/–	Non-mutated	Non-mutated	Yes	205.6	FOD
GG31	14	f	temporal	Complete	V600E	No	Negative	Non-mutated	L644L	No	104.8	FOD
GG19	6	f	posterior fossa	Complete	Non-mutated	No	+/–	Non-utated	Non-mutated	No	8.2	NA
GG07	1	f	brainstem	Complete	Non-mutated	No	Negative	Non-mutated	Non-mutated	No	130.4	AWSD
GG18	13	f	third ventricle	Partial	V600E	No	Negative	Non-mutated	Non-mutated	Yes	134.6	AWSD
GG16	12	f	frontal	Complete	Non-mutated	No	Negative	Non-mutated	Non-mutated	No	97.8	FOD
PA14	9	m	cerebellum	Complete	Non-mutated	No	Negative	Non-mutated	Non-mutated	No	64.9	FOD
PA19	3	m	cerebellum	NA	V600E	No	NA	Non-mutated	NA	No	132.5	FOD
PA11	9	m	cerebellum	Complete	V600E	No	Negative	Non-mutated	Non-mutated	No	15.4	FOD
PA01	4	m	cerebellum	Complete	Non-mutated	No	Negative	NA	NA	Yes	190.8	AWSD
PA21	4	m	cerebellum	Partial	Non-mutated	No	NA	Non-mutated	Non-mutated	Yes	59.3	FOD

Phospho-FGFR1 expression was recorded as “Positive” when all oligo-like cells were immunostained and neurons remained negative, “Negative” when no cell expressed p-FGFR1 and “+/–” when a focal expression of the phospho-protein was observed (< 20%).

Abbreviations: AWSD: alive with stable disease; DDPCR™: droplet digital PCR; DNT: dysembryoplastic neuroepithelial tumor; f: female; FOD: free of disease; GG: ganglioglioma; IHC: immunohistochemistry; m: male; NA: not available; PA: pilocytic astrocytoma; PDA: pediatric diffuse astrocytoma; PTO: pediatric-type oligodendroglioma;

* cases submitted to RNA-seq.

**Figure 1:**
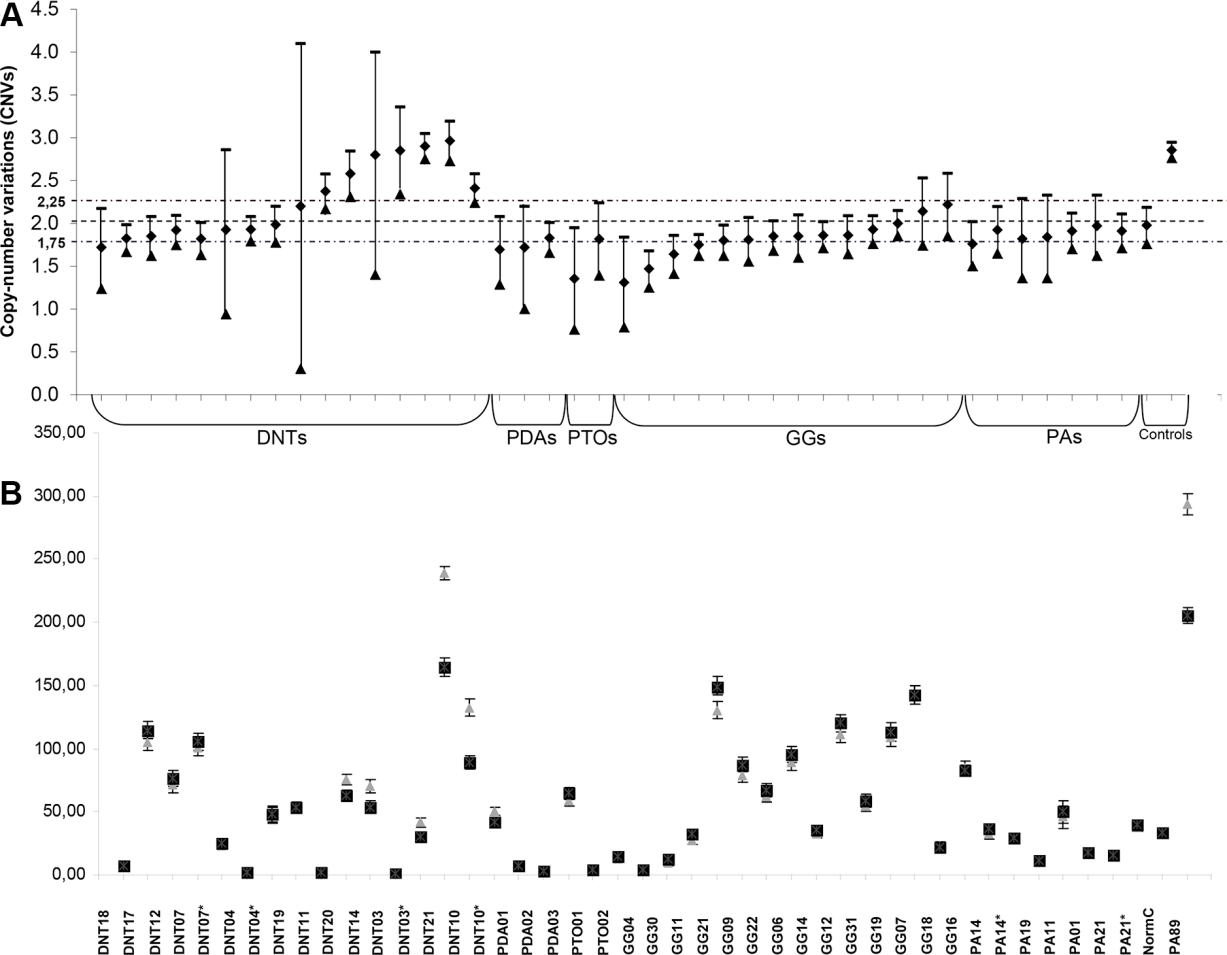
(**A**) Determination of copy number variation states of FGFR1 exon 16 compared to FGFR1 exon 8 for the 36 FFPE cases and 6 frozen additional samples (*), the placenta sample (normal control) and the positive control (PA89). When replicates were done, CNV value was the mean of replicates (additional table). Otherwise, CNV measurement was performed from a single DDPCR™ well of around 15 000 droplets. Error bars indicate the Poisson 95% confidence intervals for each DNA copy number determination. For the cases (#PTO01, #GG30; #GG04) without duplication but with low CNV value (that could demonstrate a deletion for *FGFR1*) it is worth noting that the DNA level was particularly low, making conclusion difficult. (**B**) Determination of the absolute quantification of *FGFR1* exon 16 (grey triangle) and *FGFR1* exon 8 (black square) in copies/μl for the 36 FFPE cases and 6 frozen additional samples (*), the placenta sample (normal control) and the positive control (PA89). Error bars indicate the Poisson 95% confidence intervals for each DNA copy number determination. Abbreviations: DNTs: dysembryoplastic neuroepithelial tumors; GGs: gangliogliomas, PAs: pilocytic astrocytomas; PDAs: pediatric diffuse astrocytomas; PTOs: pediatric-type oligodendrogliomas;

### FGFR1 duplication status

A total of 7177 to 47840 (median: 15691) accepted droplets were analyzed per assay (Supplementary Table S1). For each case, the cumulative droplets of several assays were combined, with a maximum total droplet count of 172031. Among the 36 FFPE cases, calculated CNV and/or CNV min values allowed for a definite assignment of copy number in 83.4% of the cases (30/36). For 5 cases, additional analyzed material was needed to determine *FGFR1* duplication status and only one case of DNT (#DNT11) remained inconclusive (Supplementary Table S1). It is important to note that when several experiments were conducted for the same patient, inter-assay reproducibility was very good and gave the same *FGFR1*duplication-status (Supplementary Figure S1 and Supplementary Table S1).

CNV reflecting *FGFR1* duplication was recorded in 5/12 DNT (41.7%) (Table 1, Figure 3 and Supplementary Table S1). Interestingly, macrodissection of one complex DNT harboring *FGFR1* duplication (#DNT21) showed that both GNE and glial nodules demonstrated duplication but not the cortex. In this series, no *FGFR1* duplication was detected in PDA, PTO, GG nor PA, therefore *FGFR1* duplication was significantly correlated to the diagnosis of DNT (*p* = 0.009). Moreover, these 5 cases did not display *BRAF* mutation (Table 1). In five cases, (#PTO01, #GG04, #GG30, #GG11 and #GG21), CNV values were low with a CNVmax under 2. However, for three out of five cases (#PTO01, #GG30, #GG04) the DNA concentration of these cases was particularly low and we cannot extrapolate if these low CNV values might reflect a *FGFR1* deletion.

One out of 5 cases displaying *FGFR1* duplication by DDPCR^TM^ was analyzed by RNA-sequencing (#DNT10) thanks to frozen material available. RNA-seq data gave clear evidence of *FGFR1*-ITD for #DNT10. Moreover, in two non-duplicated cases (#GG04 and #GG11) tested as control, RNA-seq data confirmed absence of *FGFR1*-ITD in these cases.

### Phospho-FGFR1 immunohistochemistry (Table 1)

By using an antibody directed against p-FGFR1, we observed strong immunoreactivity in DNT samples associated with *FGFR1* duplication (for 1 case, #DNT03, tissue was no longer available). The immunoreactivity was restricted to the glial compartment, especially the oligodendroglial-like cells in the GNE and in the glial nodules. Floating neurons were negative (Figure 4A and 4B). However, the intensity of the immunoreactive nuclei varied from one cell to another and one case to another, leading to difficulties in assessing the immunostaining in some cases. Therefore, although in this series p-FGFR1 immunoreactivity was correlated with DDPCR^TM^, we do not recommend p-FGFR1 immunostaining to assess *FGFR1*-ITD status. In addition to these cases we also observed less than 20% of immunopositive glial cells in another DNT (#DNT18), in the two PTOs and in 5 GGs. Dysplastic neurons always lacked p-FGFR1 expression. All PAs included in our series were negative. There was a very good correlation between *FGFR1* duplication status and “positive” p-FGFR1 immunostaining (*p* < 0.0001).

### FGFR1 mutation status

For exon 14, we reported one somatic silent mutation in one case of GG (#GG31). For exon 12, we also reported one somatic silent mutation in one case of GG (#GG14) and a somatic missense mutation which was identified in one case of DNT (#DNT20): *FGFR1*^G539R^. Interestingly, this sample also displayed *FGFR1* duplication and a strong immunoreactivity of p-FGFR1.

## DISCUSSION

In this study, we report the detection of *FGFR1* internal duplication by DDPCR™ in 5/12 DNT cases. In addition to *FGFR1* duplication, one case (#DNT20) also demonstrated a *FGFR1* point mutation (*FGFR1*^G539R^), and 3 DNT cases, 1 PTO, 2 PDAs, 5 GGs and 2 PAs displayed *BRAF*^V600E^ mutation, a feature that we have previously reported [7]. Importantly, we observed that *FGFR1* duplication was never associated with *BRAF*^V600E^ mutation, suggesting that in DNTs, as previously reported in PAs and in other LGNTs in children, these alterations are mutually exclusive [10, 11]. Altogether, more than 66% of the DNTs reported in this series displayed an alteration in the MAP Kinase pathway. Our results are in keeping with a comprehensive recent publication dedicated to a large LGNT series studied by whole-genome sequencing on frozen specimens [12].

**Figure 2:**
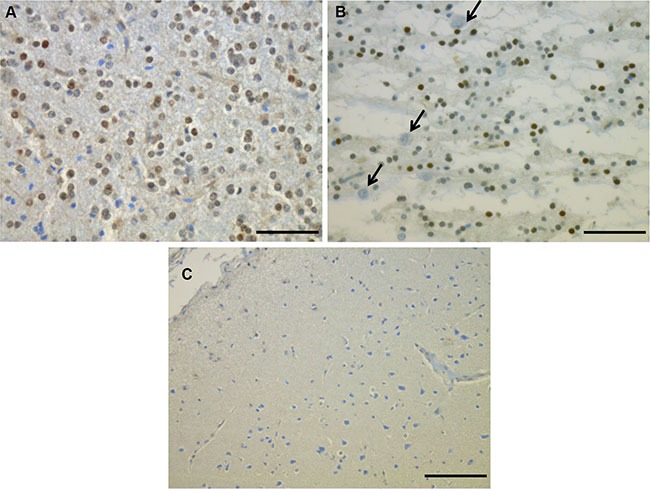
Phospho-FGFR1 protein expression detected by immunohistochemistry on two DNT cases: the immunoreactivity was restricted to the glial compartment (A and B) especially the oligodendroglial-like cells in the glial nodule (A) and the GNE (B). Floating neurons were negative (B, arrows), as well as cortex (C). Scale bars: 50 μm.

The work published by Jones and coworkers and Zhang and coworkers respectively, have shown that *FGFR1* duplication encompassed exons 10–18 to produce an in-frame fusion separated by a linker element of variable length [10, 11]. Here, we used DDPCR™ to search for *FGFR1* duplication in a large series of FFPE LGNTs. DDPCR™ utilizes sequential limiting dilutions of target DNA, followed by amplification using the polymerase chain reaction (PCR). As a result it is possible to quantitate single DNA-target molecules. The absolute quantification is measured in number of copies per μl for each single DNA-target molecule and the threshold for an amplifying signal is 5 copies per μl. This required very low amount of tissue (4 × 5 μm thick sections of FFPE tissue samples measuring 5 mm in diameter were sufficient in our experiments). Moreover, it did not require the measurement of the DNA from tissues as far as the DNA obtained was of enough quality to generate an amplifying signal. We designed probes to measure and calculate CNV for *FGFR1* exon 16 (included in the duplication of the TKD of *FGFR1*) compared to exon 8 (not involved in the duplication). CNV determination on the DDPCR™ is based upon its ability to partition DNA sequences [13]. Because we first showed that this approach was successful in one case of PA (#PA89) known to harbor the *FGFR1*-ITD, we studied the whole series and were able to detect this duplication in 5/12 DNTs. Importantly, all *FGFR1* duplicated cases also demonstrated strong p-FGFR1 accumulation by immunohistochemistry, providing additional confirmation of our results, but false positive results were also obtained. The duplication was also confirmed by RNA-seq data in one duplicated case (#DNT10) and was not found in two non-duplicated cases (#GG04 and #GG11). It is worth noticing that RNA-seq was also performed in these two last cases because of a CNVmax under 1.5 and we wondered if this could correspond to a *FGFR1:TACC1* fusion; RNA-sequencing ruled out this hypothesis. Therefore, a low CNVmax does not correspond to a *FGFR1:TACC1* fusion but likely to an artefact. However, because RNA-seq was not performed in the other cases (no frozen specimen available), the *FGFR1:TACC1* fusion status of the present cohort remains unknown.

DDPCR™ represents a powerful single molecule counting strategy to detect minute amounts of genetic material with performance surpassing many quantitative methods. This technique was successfully used in our department for the detection of several molecular alterations in different diseases [14–16]. In the present study, we took advantage of DDPCR™ to measure *FGFR1* duplication because it allows the measuring of low-amplitude CNV [17] and the accurate counting of alleles from DNA isolated from a mixture of heterogeneous cell populations and from highly degraded DNA prepared from FFPE tissue samples [18]. Previous studies have shown a very high level of concordance between DDPCR™ and exome sequencing [19] or multiplex ligation-dependent probe amplification (MLPA) [20, 21] to measure CNV. In contrast, CGH array method is not sensitive enough to detect certain CNVs, as well as FISH technique, especially if the duplicated region does not exceed 5 kb (which is the size of intragenic *FGFR1* duplication reported by Zhang et al. [11]). Moreover, literature suggests < 70% reproducibility in replicate experiments between platforms and algorithms for CGH and SNP-arrays used for CNV detection [22]. Therefore, it is not surprising that we were not able to detect this alteration previously by using CGH-array [5] and we did not try using FISH technique for the reasons mentioned above. In a recent study [23], the authors have reported frequent gains at chromosomes 5 and 7 in a large series of DNTs but they did not observe chromosome 8p11 alterations (where the *FGFR1* gene is located). It is likely that the technique used allowed them to point out large deletions/duplications but not small duplications as reported here for *FGFR1*.

To conclude, *FGFR1* duplication involving the TKD domain frequently occurs in DNTs and can be easily detected by DDPCR™ on FFPE specimen. We recommend searching for *FGFR1* aberrations and *BRAF* mutations in LGNTs in routine practice. These alterations induce upregulation of the MAP kinase pathway, which could be blocked by specific inhibitors as part of a therapeutic strategy.

## MATERIALS AND METHODS

### Patients, clinical data and pathology material

Twelve patients with specific-form of dysem bryoplastic neuroepithelial tumor (DNT) were included in this retrospective study. Two cases of pediatric-type oligodendroglioma (PTO), 3 cases of pediatric diffuse astrocytoma (PDA), 14 cases of ganglioglioma (GG) and 5 cases of pilocytic astrocytoma (PA) were studied. All DNTs, PTOs and PDAs lacked *IDH* mutation and 1p/19q co-deletion [5]. Excepting one case of DNT (#DNT21), all these patients have been described in a previous report focused on *BRAF*^V600E^ mutation detection in these tumors [7]. In this previous study, PTOs and PDAs were denominated as non-specific forms of DNT (correspondence between present and previous data: #PDA01=#DNT01; #PDA02=#DNT09; #PDA03=#DNT08; #PTO01=#DNT13; #PTO02=#DNT16).

Formalin-fixed paraffin-embedded (FFPE) pathological specimens were available in all cases and frozen samples were available for 6 cases (4 DNT and 2 PA) (AP-HM tumor bank; authorization number AC-2013-1786).

### Genomic DNA extraction and molecular analysis

Areas of viable and representative tumor following review of all blocks were marked by a pathologist (DFB). Then, tumor DNA was extracted from 4 × 5 μm thick sections of FFPE tissue samples after dewaxing (*n* = 36), and from frozen specimen (*n* = 6), as previously described [7]. The diameter of the FFPE sections varied from 5 mm to 2 cm. For one case (#DNT21), we have macrodissected the three different areas of this complex DNT: the GNE, the glial nodules and the cortex.

### BRAF^V600E^ status

In a previous study [7], we reported *BRAF* mutation status for 35 patients of the present study, by HRM-sequencing (exon 15) and/or immunohistochemistry (VE1 clone). Regarding the additional case of DNT #21, *BRAF* mutation status was analyzed by HRM-sequencing as previously described [7].

### FGFR1 duplication status

*FGFR1*-ITD recently described in LGNTs [10–12] is characterized by a duplication of the TKD of *FGFR1*. In order to search for a duplication of the TKD of the *FGFR1* gene in DNT and other LGNTs we developed droplet digital PCR (DDPCR™, Bio-rad). Therefore, we designed our assay to quantify DNA copy number of the TKD of *FGFR1* (exon 16) compared to DNA copy number of *FGFR1* exon 8 which is not affected by the duplication (Figure 1). These analysis were done for all FFPE (*n* = 36) and frozen specimen (*n* = 6). In addition, 19 cancer cell lines (colon cancer: HT29, SW48; breast cancer: BT20, MCF-7, MDAMB231, MDAMB361, SKBR3, T47D; lung cancer: A549, Calu-6, H1650; sarcoma: SW1353, H1080; leukemia: NALM6, HL60, KASUMI-1, ML-2 and melanoma: IGR37) and placenta sample (normal control) were used as negative controls whereas ICGC_PA89 sample previously published as harboring the *FGFR1-*ITD [10] was used as positive control.

In DDPCR™, target DNA molecules are distributed across multiple replicate reactions at a level where there are some reactions that have no template and others that have one or more template copies present. After amplification to the terminal plateau phase of PCR, reactions containing one or more templates yield positive end-points, whereas those without template remain negative. The number of positive and negative droplets in each reaction is used to calculate the concentration of the target and reference DNA sequences and their Poisson-based 95% confidence interval [24].

DDPCR™ experiment was performed as follows: Each 21-μl reaction mixture contained 5 μL of DNA template, 2X DDPCR™ supermix for probes (no dUTP) and FGFR1 exon 16 and exon 8 assays (Bio-Rad Laboratories). The assays were purchased as a 20X premix of primers and probes and used at 1X concentration. The 1X concentration of this assay comprised 900 nM forward primer, 900 nM reverse primer, and 250 nM probe. Primers, hydrolysis probe sequences and DDPCR™ conditions are reported in Table 2. After homogenization, the PCR reaction mixture and droplet generation oil for probes were loaded into an eight-channel droplet generator cartridge (Bio-Rad Laboratories). The PCR reaction mixtures were partitioned into an emulsion of approximately 15,000 droplets (~ 1 nL per droplet) which were manually transferred to a 96-well PCR plate. The PCR plate was heat-sealed and placed in a conventional thermal cycler. Following the PCR, the 96-well plate was loaded on a QX100 droplet reader (Bio-Rad Laboratories). Analysis of the DDPCR™ data was performed with QuantaSoft software (version 1.7.4.0917) which analyzes each droplet individually using a two-color detection system (set to detect FAM or HEX dyes).

**Table 2:** Primers, hydrolysis probes sequences and DDPCR™ conditions for the detection of *FGFR1* mutations and *FGFR1* copy number variations, reflecting *FGFR1* duplication

Primers sequences for FGFR1 mutations detection	FGFR1 Exon 12 Forward: 5′-CCTCCCTTCCCAAGTAAATGA-3′ FGFR1 Exon 12 Reverse: 5′-CTTCCCTAGCTGTGGCTGAG-3′ FGFR1 Exon 14 Forward: 5′-CTCCCTTCCTCCTTCCTCAG-3′ FGFR1 Exon 14 Reverse: 5′-ACCCCACTCCTTGCTTCTCA-3′
Primers and HEX probe sequences for Exon 8 detection	FGFR1 Exon 8 Forward: 5′-TTCCCTTGCTCTGCGTCTCT-3′ FGFR1 Exon 8 Reverse: 5′-TCCATCTCTTTGTCGGTGGTATT-3′ FGFR1 Exon 8 HEX-probe: 5′HEX-TTGCTTCCGTTGTCTCTTCTAGACTGCTGG-3′
Primers and FAM probe sequences for Exon 16 detection	FGFR1 Exon 16 Forward: 5′-CACTGCCCTGGGTAGAGGATT-3′ FGFR1 Exon 16 Reverse: 5′-ACAGGAGCACCCCGAAAGA-3′ FGFR1 Exon 16 FAM-probe: 5′FAM-CTCTAACACCCTGTGGCTCTCCGCC-3′
Thermocycler conditions	95°C, 10 min (1 cycle) 94°C, 30 sec –55°C, 1 min (40 cycles) 98°C, 10 min (1 cycle) 15°C ∞ Use of a heated lid set to 105°C

**Figure 3:**
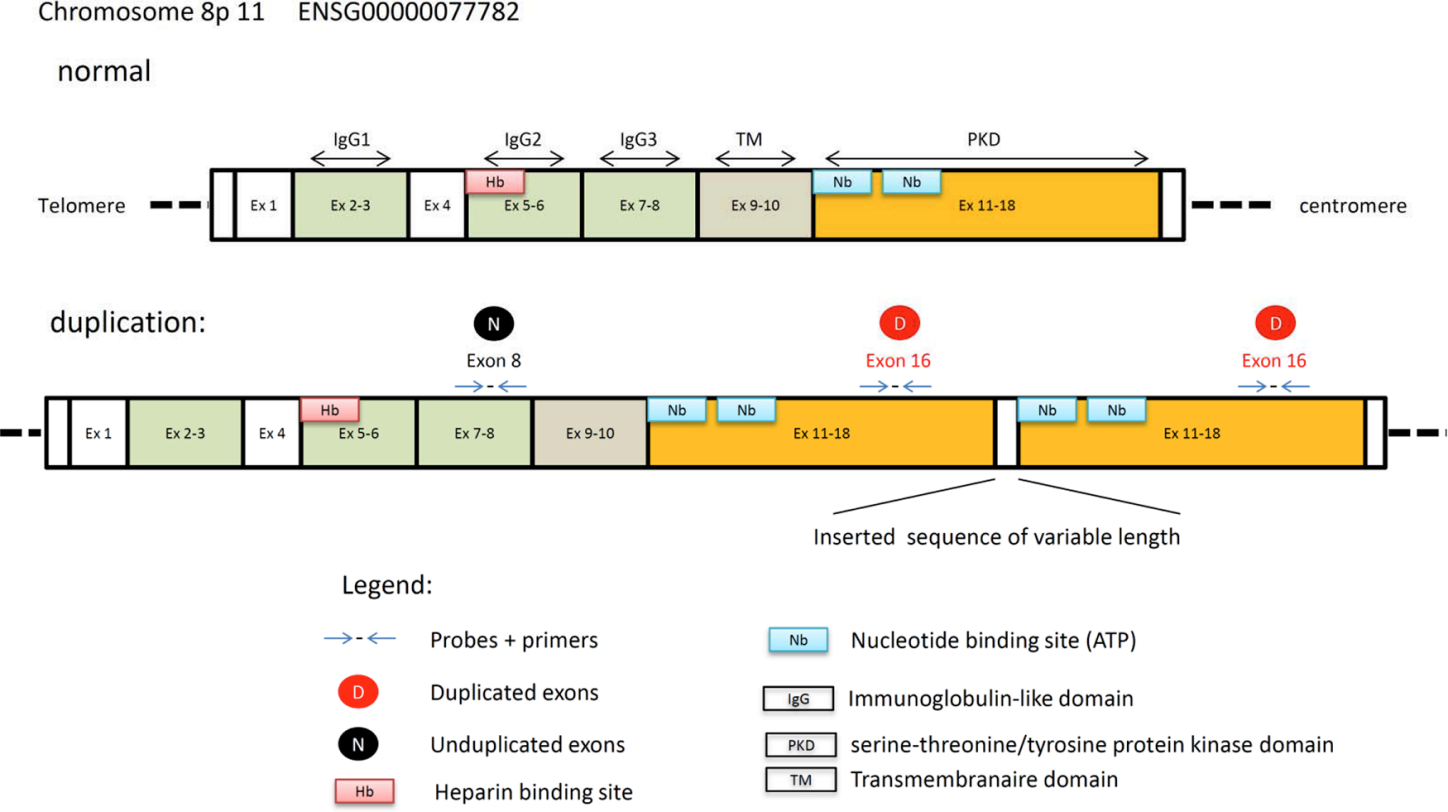
Schematic representation of the normal and the duplicated *FGFR1* gene

The absolute quantification of DNA is directly dependent on the number of accepted droplets (positive plus negative) and the DNA quantity analyzed. It requires however at least 5 copies per μl of the DNA-target molecule to allow us to give a conclusive result (see Supplementary Table S1). It is then possible to calculate the copy number variation (CNV) which represents for a haploid genome the ratio of target-DNA to reference-DNA multiplied by two. The calculation of the 95% confidence interval given by the Poisson law and the distribution of the CNV values according to our cohort of 36 samples, 19 cell lines, placenta normal control and PA89 positive control led us to consider a sample as duplicated if the CNV valuewas above 2.25 (Figure 2A) and the CNV min above 2 (Figure 2B). This corresponds to a ratio value of 1.125 for exon 16 relative to exon 8, and means theoretically a monoallelic duplication in 25% of the analyzed cells. Importantly, in case of a low DNA concentration (but sufficient amount of volume), it is possible to combine the analysis of several wells to increase the number of droplets analyzed and therefore reduce the difference between CNV max and CNV min (Supplementary Table S1).

**Figure 4:**
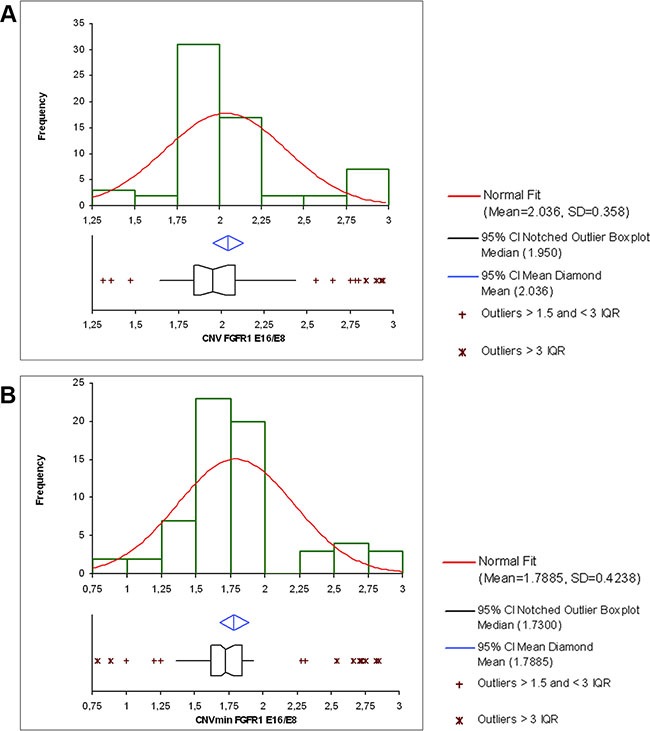
(**A**) Distribution of the CNV values for the 36 FFPE samples studied the 19 cancer cell lines, the placenta sample (normal control) and the PA89 positive control. The CNV distribution shows very few intermediate values, reflecting the powerful capacity of the droplet digital PCR to discriminate duplicated samples (2.75-3) from non-duplicated samples (1.75-2.25). The cut-off value of the CNV reflecting *FGFR1* duplication that we chose was CNV > 2.25. (**B**) Distribution of the CNV min values for the 36 FFPE samples studied, the 19 cancer cell lines, the placenta sample (normal control) and the PA89 positive control. The cut-off value of the CNV min reflecting *FGFR1* duplication that we chose was CNV min > 2.

### FGFR1 mutation status

*FGFR1* mutation status in exon 12 and exon 14 was analyzed by HRM-sequencing as previously described [7]. Point mutations were sequenced using primer pairs listed in Table 2. These analyses were done for 27/36 FFPE specimen (DNA was no longer available for the other cases). The placenta sample was used as normal control. ICGC_PA69, PA80 and PA92 samples harboring *FGFR1*^N546K^ mutation were used as positive control for mutations in exon 12 whereas ICGC_PA41 sample harboring *FGFR1*^K6551^ and *FGFR1*^K656E^ mutations, and PA84 sample displaying *FGFR1*^K656E^ mutation, were used as positive control for mutation in exon 14. These 5 additional PA samples were previously published by the ICGC PedBrain Tumor project [10].

### RNA-sequencing

In 3 cases with enough frozen material available and containing tumor tissue in more than 90%, RNA-sequencing was performed in Heidelberg as previously described [10].

### Phospho-FGFR1 immunohistochemistry

Phospho-FGFR1 (Y653/Y654) protein expression was assessed by immunohistochemistry, using antibody PA5-12594 (Thermo Scientific), as previously described [10]. This analysis was performed on 33/36 FFPE cases (3 tissue-blocks were totally spent), on 5 μm sections of tissue using an automated immunohistochemical procedure on Ventana Benchmark devices.

### Statistical analysis

The chi-square test (or Fischer's exact test, as appropriate) was used to correlate the following variables: histological diagnosis and CNV results (*FGFR1* duplication), *FGFR1* duplication and p-FGFR1 immunostaining. The tests were two-sided and the statistical significance was defined as *p* < 0.05. Analyses were conducted using PASW Statistics version 17.02 (IBM SPSS Inc., Chicago, IL, USA).

## SUPPLEMENTARY MATERIALS FIGURE AND TABLE





## References

[1] Daumas-Duport C, Scheithauer BW, Chodkiewicz JP, Laws ER, Vedrenne C (1988). Dysembryoplastic neuroepithelial tumor: a surgically curable tumor of young patients with intractable partial seizures. Report of thirty-nine cases Neurosurgery.

[2] Fernandez C, Girard N, Paz Paredes A, Bouvier-Labit C, Lena G, Figarella-Branger D (2003). The usefulness of MR imaging in the diagnosis of dysembryoplastic neuroepithelial tumor in children: a study of 14 cases. AJNR Am J Neuroradiol.

[3] Louis DN, Perry A, Reifenberger G, von Deimling A, Figarella-Branger D, Cavenee WK, Ohgaki H, Wiestler OD, Kleihues P, Ellison DW (2016). The 2016 World Health Organization Classification of Tumors of the Central Nervous System: a summary. Acta Neuropathol.

[4] Louis DN, Ohgaki H, Wiestler OD, Cavenee WK, Ellison DW, Figarella-Branger D, Perry A, Reifenberger G, von Deimling A (2016). World Health Organization Classification of Tumours of the Central Nervous System.

[5] Padovani L, Colin C, Fernandez C, Maues de Paula A, Mercurio S, Scavarda D, Frassineti F, Adelaide J, Loundou A, Intagliata D, Bouvier C, Lena G, Birnbaum D (2012). Search for distinctive markers in DNT and cortical grade II glioma in children: same clinicopathological and molecular entities?. Curr Top Med Chem.

[6] Louis DN, Ohgaki H, Wiestler OD, Cavenee WK, Burger PC, Jouvet A, Scheithauer BW, Kleihues P (2007). The 2007 WHO classification of tumours of the central nervous system. Acta Neuropathol.

[7] Chappe C, Padovani L, Scavarda D, Forest F, Nanni-Metellus I, Loundou A, Mercurio S, Fina F, Lena G, Colin C, Figarella-Branger D (2013). Dysembryoplastic neuroepithelial tumors share with pleomorphic xanthoastrocytomas and gangliogliomas BRAF(V600E) mutation and expression. Brain Pathol.

[8] Turner N, Grose R (2010). Fibroblast growth factor signalling: from development to cancer. Nat Rev Cancer.

[9] Parker BC, Engels M, Annala M, Zhang W (2014). Emergence of FGFR family gene fusions as therapeutic targets in a wide spectrum of solid tumours. J Pathol.

[10] Jones DT, Hutter B, Jager N, Korshunov A, Kool M, Warnatz HJ, Zichner T, Lambert SR, Ryzhova M, Quang DA, Fontebasso AM, Stutz AM, Hutter S (2013). Recurrent somatic alterations of FGFR1 and NTRK2 in pilocytic astrocytoma. Nat Genet.

[11] Zhang J, Wu G, Miller CP, Tatevossian RG, Dalton JD, Tang B, Orisme W, Punchihewa C, Parker M, Qaddoumi I, Boop FA, Lu C, Kandoth C (2013). Whole-genome sequencing identifies genetic alterations in pediatric low-grade gliomas. Nat Genet.

[12] Qaddoumi I, Orisme W, Wen J, Santiago T, Gupta K, Dalton JD, Tang B, Haupfear K, Punchihewa C, Easton J, Mulder H, Boggs K, Shao Y (2016). Genetic alterations in uncommon low-grade neuroepithelial tumors: BRAF, FGFR1, and MYB mutations occur at high frequency and align with morphology. Acta Neuropathol.

[13] Dube S, Qin J, Ramakrishnan R (2008). Mathematical analysis of copy number variation in a DNA sample using digital PCR on a nanofluidic device. PLoS One.

[14] Martinez E, Silvy F, Fina F, Bartoli M, Krahn M, Barlesi F, Figarella-Branger D, Iovanna J, Laugier R, Ouaissi M, Lombardo D, Mas E (2015). Rs488087 single nucleotide polymorphism as predictive risk factor for pancreatic cancers. Oncotarget.

[15] Vasilev V, Daly AF, Thiry A, Petrossians P, Fina F, Rostomyan L, Silvy M, Enjalbert A, Barlier A, Beckers A (2014). McCune-Albright syndrome: a detailed pathological and genetic analysis of disease effects in an adult patient. J Clin Endocrinol Metab.

[16] Arnoux F, Fina F, Lambert N, Balandraud N, Martin M, Ouafik L, Kanaan SB, Roudier J, Auger I (2016). New BRAF (v raf murine sarcoma viral oncogene homologue B1) mutation in rheumatoid arthritis. Arthritis Rheumatol.

[17] Weaver S, Dube S, Mir A, Qin J, Sun G, Ramakrishnan R, Jones RC, Livak KJ (2010). Taking qPCR to a higher level: Analysis of CNV reveals the power of high throughput qPCR to enhance quantitative resolution. Methods.

[18] Belgrader P, Tanner SC, Regan JF, Koehler R, Hindson BJ, Brown AS (2013). Droplet digital PCR measurement of HER2 copy number alteration in formalin-fixed paraffin-embedded breast carcinoma tissue. Clin Chem.

[19] Handsaker RE, Van Doren V, Berman JR, Genovese G, Kashin S, Boettger LM, McCarroll SA (2015). Large multiallelic copy number variations in humans. Nat Genet.

[20] Cantsilieris S, Western PS, Baird PN, White SJ (2014). Technical considerations for genotyping multi-allelic copy number variation (CNV), in regions of segmental duplication. BMC Genomics.

[21] de Smith AJ, Walsh KM, Hansen HM, Endicott AA, Wiencke JK, Metayer C, Wiemels JL (2015). Somatic Mutation Allelic Ratio Test Using ddPCR (SMART-ddPCR): An Accurate Method for Assessment of Preferential Allelic Imbalance in Tumor DNA. PLoS One.

[22] Marques FZ, Prestes PR, Pinheiro LB, Scurrah K, Emslie KR, Tomaszewski M, Harrap SB, Charchar FJ (2014). Measurement of absolute copy number variation reveals association with essential hypertension. BMC Med Genomics.

[23] Prabowo AS, van Thuijl HF, Scheinin I, Sie D, van Essen HF, Iyer AM, Spliet WG, Ferrier CH, van Rijen PC, Veersema TJ, Thom M, Schouten-van Meeteren AY, Reijneveld JC (2015). Landscape of chromosomal copy number aberrations in gangliogliomas and dysembryoplastic neuroepithelial tumours. Neuropathol Appl Neurobiol.

[24] Hindson BJ, Ness KD, Masquelier DA, Belgrader P, Heredia NJ, Makarewicz AJ, Bright IJ, Lucero MY, Hiddessen AL, Legler TC, Kitano TK, Hodel MR, Petersen JF (2015). High-throughput droplet digital PCR system for absolute quantitation of DNA copy number. Anal Chem.

