# Supplementary material for: Droplet digital PCR is a powerful technique to demonstrate frequent FGFR1 duplication in dysembryoplastic neuroepithelial tumors
Source: Oncotarget. 2016 Oct 25;8(2):2104–13. doi: 10.18632/oncotarget.12881 (PMC5356784; doi:10.18632/oncotarget.12881)
Supplement: Supplementary file 1 [file oncotarget-08-2104-s001.pdf]

Droplet digital PCR is a powerful technique to demonstrate frequent *FGFR1* duplication in dysembryoplastic neuroepithelial tumors

Supplementary Materials

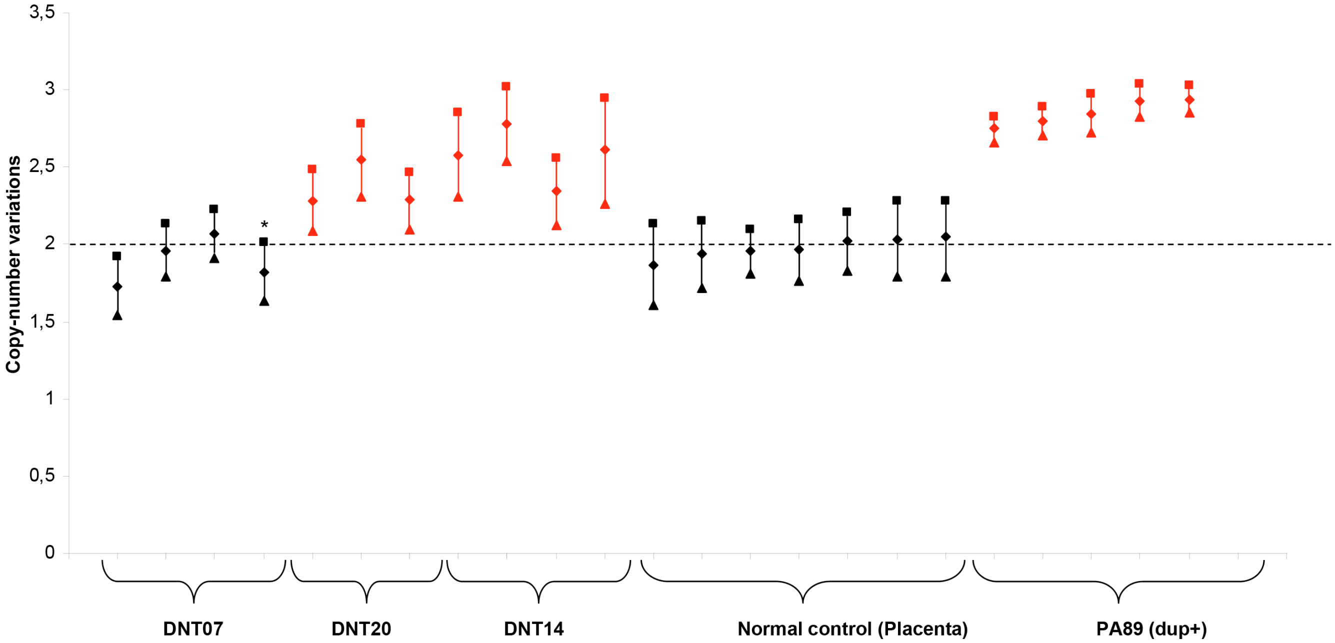

Supplementary Figure S1: DDPCR™ inter-assays reproducibility.

Supplementary Table S1: Details of DDPCR™ results and CNV calculation for all cases (FFPE tissues and *frozen tissues) to determine FGFR1 duplication status. See Supplementary_Table_S1.
