# Supplementary material for: Droplet digital PCR is a powerful technique to demonstrate frequent FGFR1 duplication in dysembryoplastic neuroepithelial tumors
Source: Oncotarget. 2016 Oct 25;8(2):2104–13. doi: 10.18632/oncotarget.12881 (PMC5356784; doi:10.18632/oncotarget.12881)
Supplement: Supplementary file 2 [file oncotarget-08-2104-s002.doc]

| **Identification** | **Conc**  **FGFR1**  **exon 16 (copies/µL)** | **Confmax**  **FGFR1 exon 16 (copies/µL)** | **Confmin**  **FGFR1 exon 16 (copies/µL)** | **Conc**  **FGFR1 exon 8 (copies/µL)** | **Confmax FGFR1 exon 8 (copies/µL)** | **Confmin FGFR1 exon 8 (copies/µL)** | **CNV FGFR1 Exon 16 over exon 8** | **CNVmax Exon 16 over exon 8** | **CNVmin exon 16 over exon 8** | **ΔEC max CNV-ΔEC min CNV** | **FGFR1 Accepted**  **Droplets** | **Dedicated wells** | **FGFR1 status** | |
| --- | --- | --- | --- | --- | --- | --- | --- | --- | --- | --- | --- | --- | --- | --- |
| DNT18 | 5,9 | 7,7 | 4,5 | 7,3 | 9,2 | 5,7 | 1,6 | 2,2 | 1 | 1,2 | 9829 | 1 | normal |  |
| DNT18 | 7,8 | 8,9 | 6,6 | 8,4 | 9,4 | 7,5 | 1,84 | 2,15 | 1,47 | 0,68 | 39159 | 3 | normal |  |
|  |  |  |  |  |  |  |  |  |  |  |  |  |  |  |
| DNT17 | 104 | 111 | 96 | 114 | 122 | 106 | 1,82 | 2 | 1,64 | 0,36 | 8116 | 1 | normal |  |
| DNT17 | 106 | 111 | 100 | 115 | 121 | 109 | 1,83 | 1,97 | 1,69 | 0,28 | 14785 | 1 | normal |  |
|  |  |  |  |  |  |  |  |  |  |  |  |  |  |  |
| DNT12 | 71 | 77 | 65 | 77 | 83 | 70 | 1,85 | 2,08 | 1,62 | 0,46 | 10660 | 1 | normal |  |
|  |  |  |  |  |  |  |  |  |  |  |  |  |  |  |
| DNT07 | 95 | 103 | 87 | 110 | 118 | 102 | 1,73 | 1,92 | 1,54 | 0,38 | 7177 | 1 | normal |  |
| DNT07 | 100 | 106 | 93,9 | 102 | 108 | 95,8 | 1,96 | 2,13 | 1,79 | 0,34 | 11982 | 1 | normal |  |
| DNT07 | 108 | 114 | 102 | 104 | 110 | 99 | 2,07 | 2,23 | 1,91 | 0,32 | 14608 | 1 | normal |  |
| DNT07* | 22,8 | 24,5 | 21,1 | 25 | 26,8 | 23,2 | 1,82 | 2,01 | 1,63 | 0,38 | 36733 | 3 | normal |  |
|  |  |  |  |  |  |  |  |  |  |  |  |  |  |  |
| DNT04 | 1,62 | 2,64 | 0,911 | 1,86 | 2,93 | 1,09 | 1,75 | 3,02 | 0,477 | 2,543 | 9480 | 1 | trends normal |  |
| DNT04 | 3,1 | 4 | 2,2 | 3 | 3,7 | 2,2 | 2,1 | 2,7 | 1,4 | 1,3 | 40528 | 3 | normal |  |
| DNT04* | 47 | 53 | 41 | 48,6 | 54 | 42 | 1,93 | 2,08 | 1,79 | 0,29 | 36401 | 3 | normal |  |
|  |  |  |  |  |  |  |  |  |  |  |  |  |  |  |
| DNT19 | 52 | 56,1 | 47,9 | 52,9 | 57,1 | 48,8 | 1,96 | 2,18 | 1,75 | 0,43 | 13478 | 1 | normal |  |
| DNT19 | 54,9 | 59,1 | 50,8 | 54,7 | 58,8 | 50,6 | 2,01 | 2,22 | 1,8 | 0,42 | 15078 | 1 | normal |  |
|  |  |  |  |  |  |  |  |  |  |  |  |  |  |  |
| DNT11 | 1,9 | 3,3 | 1 | 1,7 | 3,1 | 0,9 | 2,2 | 4,1 | 0,3 | 3,8 | 10641 | 1 | inconclusive |  |
|  |  |  |  |  |  |  |  |  |  |  |  |  |  |  |
| DNT20 | 87,5 | 92,6 | 82,5 | 76,7 | 81,4 | 72 | 2,28 | 2,48 | 2,09 | 0,39 | 15153 | 1 | duplication |  |
| DNT20 | 48,4 | 51 | 45,9 | 42,3 | 44,3 | 40,2 | 2,29 | 2,47 | 2,1 | 0,37 | 47840 | 3 | duplication |  |
| DNT20 | 90 | 95 | 85 | 70,7 | 75,5 | 65,9 | 2,55 | 2,78 | 2,31 | 0,47 | 14180 | 1 | duplication |  |
|  |  |  |  |  |  |  |  |  |  |  |  |  |  |  |
| DNT14 | 27,3 | 31,2 | 23,5 | 23,3 | 27,9 | 18,7 | 2,35 | 2,56 | 2,12 | 0,44 | 43777 | 3 | duplication |  |
| DNT14 | 106 | 114 | 99 | 82 | 89 | 76 | 2,58 | 2,85 | 2,31 | 0,54 | 8728 | 1 | duplication |  |
| DNT14 | 44,2 | 48 | 40,3 | 33,9 | 37,3 | 30,5 | 2,61 | 2,95 | 2,26 | 0,69 | 13519 | 1 | duplication |  |
| DNT14 | 105 | 110 | 99 | 75,2 | 80,1 | 70,2 | 2,78 | 3,02 | 2,54 | 0,48 | 14330 | 1 | duplication |  |
|  |  |  |  |  |  |  |  |  |  |  |  |  |  |  |
| DNT03 | 1,38 | 1,8 | 1,04 | 0,98 | 1,33 | 0,62 | 2,8 | 4 | 1,4 | 2,6 | 43436 | 3 | trends duplication |  |
| DNT03* | 53,8 | 58,4 | 49,4 | 40,7 | 44,4 | 36,9 | 2,65 | 3,02 | 2,28 | 0,74 | 41720 | 3 | duplication |  |
| DNT03* | 29,2 | 32 | 26,4 | 19,2 | 22,1 | 16 | 3,05 | 3,7 | 2,4 | 1,3 | 32946 | 3 | duplication |  |
|  |  |  |  |  |  |  |  |  |  |  |  |  |  |  |
| DNT21 | 239 | 244 | 234 | 164,7 | 172 | 157 | 2,9 | 3,05 | 2,75 | 0,3 | 44434 | 3 | duplication |  |
|  |  |  |  |  |  |  |  |  |  |  |  |  |  |  |
| DNT10 | 74,1 | 77,5 | 70,7 | 61,1 | 63,7 | 58,5 | 2,43 | 2,56 | 2,28 | 0,28 | 42344 | 3 | duplication |  |
| DNT10 | 149 | 157 | 141 | 118 | 125 | 111 | 2,53 | 2,74 | 2,32 | 0,42 | 10988 | 1 | duplication |  |
| DNT10 | 174 | 183 | 165 | 89 | 95 | 82 | 3,93 | 4,28 | 3,58 | 0,7 | 9473 | 1 | duplication |  |
| DNT10* | 50,7 | 53,6 | 47,6 | 42,1 | 44,2 | 39,9 | 2,41 | 2,58 | 2,24 | 0,34 | 41692 | 3 | duplication |  |
| **Identification** | **Conc**  **FGFR1**  **exon 16 (copies/µL)** | **Confmax**  **FGFR1 exon 16 (copies/µL)** | **Confmin**  **FGFR1 exon 16 (copies/µL)** | **Conc**  **FGFR1 exon 8 (copies/µL)** | **Confmax FGFR1 exon 8 (copies/µL)** | **Confmin FGFR1 exon 8 (copies/µL)** | **CNV FGFR1 Exon 16 over exon 8** | **CNVmax Exon 16 over exon 8** | **CNVmin exon 16 over exon 8** | **ΔEC max CNV-ΔEC min CNV** | **FGFR1 Accepted**  **Droplets** | **Dedicated wells** | **FGFR1 status** |  |
| PTO01 | 3,6 | 5,2 | 2,4 | 5,5 | 7,4 | 4 | 1,3 | 2 | 0,7 | 1,3 | 8742 | 1 | trends deleted |  |
| PTO 01 | 2,8 | 3,4 | 2,3 | 4,2 | 5,1 | 3,2 | 1,36 | 1,75 | 0,88 | 0,87 | 42017 | 3 | deleted |  |
| PTO 01 | 2,7 | 3,8 | 1,8 | 3,8 | 5,1 | 2,7 | 1,4 | 2,1 | 0,7 | 1,4 | 11557 | 1 | trends deleted |  |
|  |  |  |  |  |  |  |  |  |  |  |  |  |  |  |
| PTO 02 | 11,9 | 14,2 | 9,6 | 15,1 | 17,7 | 12,5 | 1,58 | 1,99 | 1,17 | 0,82 | 9536 | 1 | trends normal |  |
| PTO 02 | 13,1 | 15,1 | 11,1 | 14,5 | 16,6 | 12,4 | 1,81 | 2,19 | 1,43 | 0,76 | 14879 | 1 | normal |  |
| PTO 02 | 14 | 16,4 | 11,7 | 13,6 | 15,9 | 11,4 | 2,06 | 2,54 | 1,57 | 0,97 | 11110 | 1 | normal |  |
|  |  |  |  |  |  |  |  |  |  |  |  |  |  |  |
| PDA01 | 7,1 | 8 | 6 | 7,7 | 8,6 | 6,8 | 1,84 | 2,15 | 1,49 | 0,66 | 41989 | 3 | normal |  |
|  |  |  |  |  |  |  |  |  |  |  |  |  |  |  |
| PDA02 | 2,7 | 3,5 | 1,9 | 3,1 | 3,7 | 2,5 | 1,72 | 2,2 | 1 | 1,2 | 42833 | 3 | normal |  |
|  |  |  |  |  |  |  |  |  |  |  |  |  |  |  |
| PDA03 | 60,9 | 64,9 | 56,9 | 67,6 | 71,9 | 63,4 | 1,8 | 1,96 | 1,64 | 0,32 | 16549 | 1 | normal |  |
| PDA03 | 57,2 | 61,4 | 53 | 61,4 | 65,8 | 57 | 1,86 | 2,06 | 1,67 | 0,39 | 14776 | 1 | normal |  |
|  |  |  |  |  |  |  |  |  |  |  |  |  |  |  |
| GG04 | 3.08 | 4.14 | 2.22 | 4.70 | 5.99 | 3.62 | 1.31 | 1.84 | 0.79 | 1.06 | 14283 | 1 | Trends deleted |  |
|  |  |  |  |  |  |  |  |  |  |  |  |  |  |  |
| GG30 | 9,2 | 10,6 | 7,8 | 12,6 | 13,7 | 11,4 | 1,47 | 1,68 | 1,25 | 0,43 | 44101 | 3 | deleted |  |
|  |  |  |  |  |  |  |  |  |  |  |  |  |  |  |
| GG11 | 26,9 | 29,7 | 24,1 | 32,9 | 36 | 29,8 | 1,64 | 1,86 | 1,41 | 0,45 | 15091 | 1 | deleted |  |
|  |  |  |  |  |  |  |  |  |  |  |  |  |  |  |
| GG21 | 130 | 137 | 124 | 149 | 157 | 142 | 1,75 | 1,87 | 1,62 | 0,25 | 12442 | 1 | deleted |  |
|  |  |  |  |  |  |  |  |  |  |  |  |  |  |  |
| GG09 | 78,5 | 84,1 | 72,9 | 87,2 | 93,1 | 81,3 | 1,8 | 1,98 | 1,62 | 0,36 | 10938 | 1 | normal |  |
|  |  |  |  |  |  |  |  |  |  |  |  |  |  |  |
| GG22 | 16,1 | 18,6 | 13,7 | 18,3 | 20,9 | 15,7 | 1,76 | 2,13 | 1,4 | 0,73 | 12180 | 1 | normal |  |
| GG22 | 108 | 114 | 101 | 116 | 123 | 109 | 1,86 | 2,01 | 1,71 | 0,3 | 11793 | 1 | normal |  |
|  |  |  |  |  |  |  |  |  |  |  |  |  |  |  |
| GG06 | 88,7 | 94,8 | 82,6 | 95,8 | 102 | 89,5 | 1,85 | 2,03 | 1,68 | 0,35 | 10641 | 1 | normal |  |
|  |  |  |  |  |  |  |  |  |  |  |  |  |  |  |
| GG14 | 32,8 | 36 | 29,6 | 35,5 | 38,8 | 32,1 | 1,85 | 2,1 | 1,6 | 0,5 | 13575 | 1 | normal |  |
|  |  |  |  |  |  |  |  |  |  |  |  |  |  |  |
| GG12 | 111 | 118 | 105 | 120 | 127 | 113 | 1,86 | 2,02 | 1,71 | 0,31 | 11083 | 1 | normal |  |
|  |  |  |  |  |  |  |  |  |  |  |  |  |  |  |
| GG31 | 54,7 | 59,5 | 50 | 58,8 | 63,7 | 53,9 | 1,86 | 2,09 | 1,64 | 0,45 | 10660 | 1 | normal |  |
|  |  |  |  |  |  |  |  |  |  |  |  |  |  |  |
| GG19 | 109 | 116 | 102 | 113 | 120 | 106 | 1,93 | 2,09 | 1,76 | 0,33 | 11095 | 1 | normal |  |
|  |  |  |  |  |  |  |  |  |  |  |  |  |  |  |
| GG07 | 143 | 150 | 135 | 143 | 150 | 135 | 2 | 2,15 | 1,85 | 0,3 | 11206 | 1 | normal |  |
|  |  |  |  |  |  |  |  |  |  |  |  |  |  |  |
| GG18 | 23,3 | 26,2 | 20,3 | 21,8 | 24,6 | 18,9 | 2,14 | 2,53 | 1,74 | 0,79 | 11220 | 1 | normal |  |
|  |  |  |  |  |  |  |  |  |  |  |  |  |  |  |
| GG16 | 159 | 166 | 152 | 156 | 163 | 149 | 2,04 | 2,17 | 1,9 | 0,27 | 13451 | 1 | normal |  |
| GG16 | 11,4 | 13,3 | 9,4 | 9,6 | 11,4 | 7,8 | 2,4 | 3 | 1,8 | 1,2 | 13526 | 1 | normal |  |
| **Identification** | **Conc**  **FGFR1**  **exon 16 (copies/µL)** | **Confmax**  **FGFR1 exon 16 (copies/µL)** | **Confmin**  **FGFR1 exon 16 (copies/µL)** | **Conc**  **FGFR1 exon 8 (copies/µL)** | **Confmax FGFR1 exon 8 (copies/µL)** | **Confmin FGFR1 exon 8 (copies/µL)** | **CNV FGFR1 Exon 16 over exon 8** | **CNVmax Exon 16 over exon 8** | **CNVmin exon 16 over exon 8** | **ΔEC max CNV-ΔEC min CNV** | **FGFR1 Accepted**  **Droplets** | **Dedicated wells** | **FGFR1 status** |  |
| PA14 | 32,1 | 35,5 | 28,6 | 36,4 | 40,1 | 32,7 | 1,76 | 2,02 | 1,5 | 0,52 | 11445 | 1 | normal |  |
| PA14* | 12,7 | 14,7 | 10,7 | 13,8 | 15,9 | 11,7 | 1,84 | 2,25 | 1,43 | 0,82 | 13992 | 1 | normal |  |
| PA14* | 28,3 | 30,2 | 26,3 | 30,7 | 33 | 28,3 | 1,84 | 2 | 1,68 | 0,32 | 42239 | 3 | normal |  |
| PA14* | 45,8 | 49,8 | 41,9 | 43,9 | 47,7 | 40,1 | 2,09 | 2,34 | 1,83 | 0,51 | 12876 | 1 | normal |  |
|  |  |  |  |  |  |  |  |  |  |  |  |  |  |  |
| PA19 | 10,8 | 12,8 | 8,86 | 11,9 | 14 | 9,81 | 1,82 | 2,29 | 1,36 | 0,93 | 11716 | 1 | normal |  |
|  |  |  |  |  |  |  |  |  |  |  |  |  |  |  |
| PA11 | 46 | 54 | 37 | 50 | 59 | 41 | 1,84 | 2,33 | 1,36 | 0,97 | 10938 | 1 | normal |  |
|  |  |  |  |  |  |  |  |  |  |  |  |  |  |  |
| PA01 | 17,1 | 18,4 | 15,7 | 17,9 | 19,5 | 16,3 | 1,91 | 2,12 | 1,7 | 0,42 | 42766 | 3 | normal |  |
|  |  |  |  |  |  |  |  |  |  |  |  |  |  |  |
| PA21 | 16 | 18 | 13,9 | 16,2 | 18,2 | 14,1 | 1,97 | 2,33 | 1,62 | 0,71 | 16229 | 1 | normal |  |
| PA21* | 37,6 | 40,4 | 34,8 | 39,3 | 41,3 | 37,3 | 1,91 | 2,11 | 1,71 | 0,4 | 45316 | 3 | normal |  |
|  |  |  |  |  |  |  |  |  |  |  |  |  |  |  |
| A549 | 27,1 | 29,5 | 24,7 | 25,9 | 28,7 | 23,2 | 2,1 | 2,39 | 1,78 | 0,61 | 21986 | 2 | normal |  |
|  |  |  |  |  |  |  |  |  |  |  |  |  |  |  |
| BT20 | 17,5 | 19,1 | 16,1 | 16,8 | 19,4 | 14,3 | 2,09 | 2,33 | 1,83 | 0,5 | 39532 | 3 | normal |  |
| BT20 | 12 | 14,3 | 9,9 | 10,4 | 12,3 | 8,3 | 2,31 | 3,2 | 1,5 | 1,7 | 28523 | 2 | normal |  |
|  |  |  |  |  |  |  |  |  |  |  |  |  |  |  |
| Calu-6 | 30,6 | 33,1 | 28 | 29,2 | 32,2 | 26,1 | 2,1 | 2,35 | 1,84 | 0,51 | 20831 | 2 | normal |  |
|  |  |  |  |  |  |  |  |  |  |  |  |  |  |  |
| H1080 | 18,6 | 20,5 | 16,8 | 20,8 | 22,8 | 18,9 | 1,79 | 2,03 | 1,54 | 0,49 | 24860 | 2 | normal |  |
|  |  |  |  |  |  |  |  |  |  |  |  |  |  |  |
| H1650 | 21,7 | 24,3 | 18,9 | 21,2 | 25,9 | 16,3 | 2,05 | 2,26 | 1,82 | 0,44 | 43338 | 3 | normal |  |
|  |  |  |  |  |  |  |  |  |  |  |  |  |  |  |
| HL60 | 56,2 | 59,5 | 52,9 | 59,1 | 62,5 | 55,7 | 1,9 | 2,06 | 1,75 | 0,31 | 23868 | 2 | normal |  |
|  |  |  |  |  |  |  |  |  |  |  |  |  |  |  |
| HT29 | 51 | 58 | 44 | 54 | 62 | 46 | 1,89 | 2,07 | 1,72 | 0,35 | 20436 | 2 | normal |  |
|  |  |  |  |  |  |  |  |  |  |  |  |  |  |  |
| IGR37 | 85,8 | 89,5 | 82,1 | 83,9 | 87,2 | 80,7 | 2,05 | 2,19 | 1,89 | 0,3 | 37961 | 3 | normal |  |
|  |  |  |  |  |  |  |  |  |  |  |  |  |  |  |
| KASUMI-1 | 9,6 | 11 | 8,3 | 9,2 | 10,5 | 7,9 | 2,1 | 2,52 | 1,69 | 0,83 | 24737 | 2 | normal |  |
|  |  |  |  |  |  |  |  |  |  |  |  |  |  |  |
| MCF-7 | 21,9 | 24,7 | 19,1 | 21,4 | 24,2 | 18,6 | 2,05 | 2,42 | 1,67 | 0,75 | 11917 | 1 | normal |  |
|  |  |  |  |  |  |  |  |  |  |  |  |  |  |  |
| MDAMB231 | 5,6 | 6,7 | 4,6 | 6 | 8,2 | 4 | 1,86 | 2,8 | 0,9 | 1,9 | 23085 | 2 | normal |  |
| MDAMB231 | 8,6 | 9,8 | 7,4 | 8,2 | 9,3 | 7,2 | 2,08 | 2,42 | 1,71 | 0,71 | 41368 | 3 | normal |  |
|  |  |  |  |  |  |  |  |  |  |  |  |  |  |  |
| MDAMB361 | 29,1 | 31,2 | 26,7 | 28,7 | 30,8 | 26,7 | 2,03 | 2,21 | 1,81 | 0,4 | 36476 | 3 | normal |  |
| MDAMB361 | 18,1 | 22,3 | 13,8 | 15,7 | 22 | 9 | 2,31 | 2,79 | 1,8 | 0,99 | 12324 | 1 | normal |  |
|  |  |  |  |  |  |  |  |  |  |  |  |  |  |  |
| ML-2 | 46,1 | 49,1 | 42,9 | 44,6 | 47,6 | 41,7 | 2,07 | 2,26 | 1,87 | 0,39 | 23469 | 2 | Normal |  |
|  |  |  |  |  |  |  |  |  |  |  |  |  |  |  |
| **Identification** | **Conc**  **FGFR1**  **exon 16 (copies/µL)** | **Confmax**  **FGFR1 exon 16 (copies/µL)** | **Confmin**  **FGFR1 exon 16 (copies/µL)** | **Conc**  **FGFR1 exon 8 (copies/µL)** | **Confmax FGFR1 exon 8 (copies/µL)** | **Confmin FGFR1 exon 8 (copies/µL)** | **CNV FGFR1 Exon 16 over exon 8** | **CNVmax Exon 16 over exon 8** | **CNVmin exon 16 over exon 8** | **ΔEC max CNV-ΔEC min CNV** | **FGFR1 Accepted**  **Droplets** | **Dedicated wells** | **FGFR1 status** |  |
| NALM6 | 55,2 | 62 | 49 | 54,8 | 58 | 51,6 | 2,01 | 2,2 | 1,82 | 0,38 | 24861 | 2 | normal |  |
|  |  |  |  |  |  |  |  |  |  |  |  |  |  |  |
| SW1353 | 43,2 | 47 | 39,3 | 43,5 | 47,4 | 39,7 | 1,98 | 2,23 | 1,73 | 0,5 | 13573 | 1 | normal |  |
|  |  |  |  |  |  |  |  |  |  |  |  |  |  |  |
| SW48 | 13,9 | 15,5 | 12,3 | 15,6 | 17,3 | 13,9 | 1,78 | 2,08 | 1,46 | 0,62 | 24830 | 2 | normal |  |
| SW48 | 15,5 | 17 | 14 | 15,8 | 17,9 | 13,7 | 1,96 | 2,19 | 1,73 | 0,46 | 41650 | 3 | normal |  |
| SW48 | 15,8 | 17,1 | 14,5 | 15,5 | 16,9 | 14 | 2,04 | 2,28 | 1,79 | 0,49 | 44445 | 3 | normal |  |
|  |  |  |  |  |  |  |  |  |  |  |  |  |  |  |
| T47D | 6,2 | 8 | 4,7 | 5,8 | 7,5 | 4,3 | 2,2 | 3 | 1,3 | 1,7 | 10336 | 1 | normal |  |
|  |  |  |  |  |  |  |  |  |  |  |  |  |  |  |
| THP-1 | 63,1 | 66,6 | 59,3 | 60,9 | 64,4 | 57,5 | 2,07 | 2,23 | 1,9 | 0,33 | 23908 | 2 | normal |  |
|  |  |  |  |  |  |  |  |  |  |  |  |  |  |  |
| Normal control | 38,7 | 42,6 | 34,9 | 41,4 | 45,4 | 37,5 | 1,87 | 2,13 | 1,61 | 0,52 | 11320 | 1 | normal |  |
| Normal control | 31,5 | 33,9 | 29 | 32,5 | 35,2 | 29,8 | 1,94 | 2,15 | 1,72 | 0,43 | 24260 | 2 | normal |  |
| Normal control | 39,8 | 41,9 | 37,7 | 40,7 | 42,8 | 38,6 | 1,96 | 2,1 | 1,81 | 0,29 | 43292 | 3 | normal |  |
| Normal control | 28,1 | 30,2 | 26,1 | 28,6 | 31,6 | 25,8 | 1,97 | 2,16 | 1,76 | 0,4 | 31319 | 3 | normal |  |
| Normal control | 23,3 | 24,9 | 21,8 | 23,1 | 24,8 | 21,4 | 2,02 | 2,21 | 1,83 | 0,38 | 44426 | 3 | normal |  |
| Normal control | 45 | 48,8 | 41,1 | 44,3 | 48,1 | 40,5 | 2,03 | 2,28 | 1,79 | 0,49 | 13296 | 1 | normal |  |
| Normal control | 25,3 | 27,6 | 23,2 | 24,7 | 26,4 | 23,1 | 2,05 | 2,28 | 1,79 | 0,49 | 42887 | 3 | normal |  |
|  |  |  |  |  |  |  |  |  |  |  |  |  |  |  |
| ICGC PA89 | 289 | 299 | 279 | 210 | 214,8 | 205,2 | 2,75 | 2,83 | 2,66 | 0,17 | 45279 | 3 | duplication |  |
| ICGC PA89 | 283 | 294 | 272 | 201,8 | 211 | 192 | 2,8 | 2,89 | 2,71 | 0,18 | 42071 | 3 | duplication |  |
| ICGC PA89 | 286 | 294 | 277 | 201 | 208 | 194 | 2,84 | 2,97 | 2,72 | 0,27 | 22708 | 2 | duplication |  |
| ICGC PA89 | 310 | 317 | 303 | 211 | 217 | 205 | 2,93 | 3,04 | 2,83 | 0,21 | 31112 | 3 | duplication |  |
| ICGC PA89 | 293 | 299 | 287 | 199,3 | 206 | 193 | 2,94 | 3,03 | 2,85 | 0,18 | 42974 | 3 | duplication |  |
